# More Than Meets the Eye: Income and Racial Disparities in Vision Health of Older Adults

**DOI:** 10.1093/geroni/igaa057.1097

**Published:** 2020-12-16

**Authors:** Asif Iqbal, Ananya Sarker

**Affiliations:** 1 Case Western Reserive University, Cleveland, Ohio, United States; 2 Case Western Reserve University, Cleveland, Ohio, United States

## Abstract

By the end of this decade, the USA is projected to experience an increase of more than 50% in the number of people with poorer vision health as the population grows older. Using data from the NHANES III (1988–1994) for 7,186 White, Black, and Hispanic adults of ages 50 to 90 (Mean=68.23, SD=10), this paper examines the racial/ethnic, and socioeconomic disparities in vision health in the older adult population. The focus of this paper is on Visual Impairment indicators: full/partial blindness and trouble seeing with glasses to demonstrate vision health disparities in these race/ethnic groups in terms of family income using logistic regression analysis. Controls include demographic characteristics like age, gender, marital status, region, education and behavioral features like alcohol consumption and smoking. We explore another component of vision health: days since last visit to healthcare provider to evaluate the inequalities in access to health care using OLS regression analysis. In Whites (OR=.85) and Blacks (OR=.63), people with less family income are more likely to experience blindness, however, there exists no significant variability in blindness in terms of family income among Hispanics. In Black (OR=.82) and Hispanics (OR=.85), people with less family income are more likely to have trouble seeing even with glasses, however, this relationship does not exist among Whites. Days since last visit can be explained by income for Whites (Beta=-92), not for Blacks and Hispanics. This compounded disparity puts a disproportionate economic burden on minority groups, but the current Medicare policy fails to address that.

